# Comparative Microbiome Profiles of Korean Fermented Foods Based on Production Type and Additive Use

**DOI:** 10.3390/foods15061010

**Published:** 2026-03-12

**Authors:** Huyong Lee, Woori Na, Cheongmin Sohn

**Affiliations:** 1 Institute of Life Resources and Science, Wonkwang University, 460 Iksan-daero, Iksan 54538, Jeonbuk, Republic of Korea; lhy91525@naver.com (H.L.); nawoori6@gmail.com (W.N.); 2 Department of Food and Nutrition, College of Health and Welfare, Wonkwang University, 460 Iksan-daero, Iksan 54538, Jeonbuk, Republic of Korea

**Keywords:** fermented foods, microbial diversity, amplicon sequencing, traditional fermentation, commercial fermentation

## Abstract

Fermented foods are produced through controlled microbial activity and are valued for their extended shelf life, sensory attributes, and potential health benefits. This study examined the effects of production methods on microbial ecology by comparing microbial community structure, Shannon diversity, and pH changes in traditional and commercially produced Korean fermented foods. Cabbage and radish kimchi were fermented for four weeks to assess microbial succession and physicochemical changes, and additional fermented foods, including soy sauce, soybean paste, pepper paste, fruit vinegar, yogurt, and aged kimchi, were compared according to production method. Microbial communities were analyzed using amplicon sequencing targeting the V3–V4 regions of the bacterial 16S rRNA gene and the fungal internal transcribed spacer (ITS) region. Traditionally produced cabbage kimchi exhibited high microbial diversity at the early fermentation stage, initially dominated by *Weissella* and *Leuconostoc*, followed by a gradual shift toward lactic acid bacteria dominance at later stages. In contrast, commercially produced cabbage kimchi maintained a simplified microbial community dominated by a limited number of lactic acid bacteria throughout fermentation. Radish kimchi showed production-method-dependent patterns, with the rapid dominance of lactic acid bacteria during traditional fermentation and partial recovery of microbial diversity during commercial fermentation. Shannon diversity was consistently higher in traditionally produced kimchi during fermentation. In contrast, commercially produced kimchi exhibited more rapid acidification. Across other fermented foods, traditionally produced soy-based products exhibited complex microbial communities dominated by *Bacillus* spp., whereas commercially produced products were characterized by yeast-dominant profiles. Fruit vinegar and yogurt showed low microbial diversity regardless of the production method. These findings demonstrate the importance of production strategies in shaping microbial ecology, fermentation dynamics, and resulting product characteristics across various Korean fermented foods.

## 1. Introduction

Fermented foods are produced by controlling the growth of microorganisms in foods and have long been used to extend shelf life and to enhance characteristics such as flavor and texture [1,2]. When foods are fermented, the microorganisms involved in fermentation produce a variety of metabolites, which not only inhibit the growth of pathogenic microorganisms in foods but also can improve the sensory properties of the foods, and it benefits the health of humans who consume them [3,4]. For these reasons, fermented foods are consumed worldwide, and their consumption has increased across the globe in recent years due to growing interest in health [4]. Microorganisms from fermented foods may survive gastrointestinal transit to some extent and transiently interact with the gut microbiota, potentially exerting functional effects on the gut environment [4,5]. The gut microbiota is a community of microorganisms residing in the human gastrointestinal tract and is closely associated with food intake, contributing to human health through vitamin synthesis, fermentation, and absorption of fatty acids, suppression of pathogenic microorganisms, and modulation of the immune system [5,6,7,8,9,10,11,12].

In response to the recent increase in demand for fermented food consumption, commercial fermentation processes that enable large-scale production and standardized product quality have become predominant to overcome the low productivity of traditional fermentation processes [13,14]. However, previous studies have suggested that microbial diversity and microbial metabolite production may differ depending on production methods [15,16]. Although numerous studies have investigated microbial community differences in individual fermented foods produced by traditional or commercial methods or compared the two production methods, these efforts have largely focused on specific product categories [17,18,19,20,21]. For example, differences in microbial communities have been reported for traditionally and commercially produced fermented sausages [22], kefir [17], feta cheese [20], soy sauce [21], and kimchi [18]. Nevertheless, comprehensive comparisons that apply the same microbial community analysis and diversity metrics across a wide range of fermented food types remain limited.

Korean fermented foods are characterized by substantial variations in microbial communities and metabolite profiles depending on ingredients and manufacturing methods [23,24,25,26], making them a suitable model system for understanding the diversity and complexity of fermentation processes. In particular, kimchi, a representative Korean fermented food, exhibits pronounced differences in microbial communities, pH, and flavor even due to variations in minor ingredients added in addition to the main raw materials, making it an ideal model for analyzing the interactions between microbial ecology and physicochemical changes over time during fermentation under traditional and commercial production conditions [27,28,29]. Such comparative studies are essential for elucidating the relationships among fermentation methods, microbial status, and associated physicochemical changes.

Therefore, the objectives of this study are twofold: (i) to compare the microbial community structures of traditional and commercial Korean fermented foods and (ii) to investigate changes in microbial diversity and pH during a four-week fermentation period of representative kimchi types commonly consumed in Korea, thereby elucidating differences in microbial ecology between traditional and commercial fermentation and providing fundamental data on the temporal dynamics of kimchi fermentation.

## 2. Materials and Methods

### 2.1. Selection of Korean Fermented Foods

This study analyzed representative Korean fermented foods selected according to classifications and guidelines provided by the Ministry of Food and Drug Safety [30] and the Ministry of Agriculture, Food and Rural Affairs [31] of the Republic of Korea. Fermented foods were grouped into kimchi products and non-kimchi fermented foods. Kimchi products included cabbage kimchi, radish kimchi, and aged kimchi, whereas non-kimchi fermented foods included soy-based products (soy sauce, soybean paste, and pepper paste), yogurt, and fruit vinegar. In addition, products were categorized based on formulation characteristics and market distribution type. Products obtained from small-scale producers and labeled as additive-free were classified as traditionally produced, whereas products containing declared food additives and widely available in retail markets were classified as commercially available products. This categorization was based on label information and regulatory definitions. Information regarding starter culture use was collected through direct communication with manufacturers and examination of product label information. Starter use was confirmed in some products, whereas it was absent or not declared in others, including within both traditionally produced and commercially available categories. Because the starter application was not consistently associated with production type, inoculation status was not used as a classification criterion in this study. Therefore, the production categories used here reflect additive use and market distribution characteristics rather than confirmed starter culture application. All commercial products were widely available consumer products selected to represent commonly consumed retail products in the Korean market. Samples were purchased within 7 days of the indicated manufacturing date to minimize additional fermentation and microbial succession during post-production storage. After purchase, products obtained from retail markets were transported directly to the laboratory on the day of purchase. Products purchased via online markets were delivered by courier; refrigerated products were shipped in insulated containers with ice packs to maintain a temperature of approximately 4 °C during transit until delivered. Upon arrival at the laboratory, all samples were immediately stored at 4 °C until microbiome analysis. Detailed information on product manufacturers, production procedures, and starter culture use is provided in Supplementary Tables S1–S6.

### 2.2. Sampling and Preparation of Fermented Foods

Fermented food samples produced by traditional and commercial methods were selected for analysis. Kimchi products included cabbage kimchi (*n* = 5), radish kimchi (*n* = 5), and aged kimchi (*n* = 5). Additional fermented foods included soy sauce (*n* = 9), soybean paste (*n* = 6), pepper paste (*n* = 7), fruit vinegar (*n* = 6), and yogurt (*n* = 5). For kimchi products, samples were collected from different parts of the products, including leaves, stems, and core tissues. Approximately 20 g from each part was aseptically collected using sterilized forceps and scissors in sterile beakers and subsequently homogenized using a homogenizer. For semi-solid fermented foods, including soybean paste and pepper paste, samples were thoroughly mixed using a sterilized spoon, after which 10 g of each sample was transferred to sterile sample bags and homogenized. Liquid fermented foods, including yogurt, soy sauce, and fruit vinegar, were thoroughly shaken to ensure uniform suspension of sediments. Subsequently, 40 mL of each sample was transferred to sterile bottles and homogenized using a vortex mixer. All samples were collected in triplicate and pooled according to fermented food type prior to downstream analyses.

### 2.3. Genomic DNA (gDNA) Extraction and Amplicon Sequencing of Bacterial and Fungal Communities

gDNA was extracted from each fermented food sample using the DNeasy PowerSoil Pro Kit (Qiagen, Hilden, Germany) according to the manufacturer’s instructions. Homogenized samples were filtered through sterile syringe filters, followed by centrifugation at 4 °C and 4000× *g* for 15 min. All sample preparations and analyses were performed in triplicate. DNA concentration was quantified using a VICTOR Nivo™ multimode plate reader (PerkinElmer, Waltham, MA, USA) with PicoGreen reagents. For microbial community analysis, amplicon sequencing was conducted by an external sequencing service provider (Macrogen, Sejong, Republic of Korea). After sequencing, adapter and primer sequences were removed using Cutadapt (v3.2), and quality filtering and amplicon sequence variant (ASV) inference were performed using the DADA2 pipeline (v1.18.0). Sequences with high expected error values, ambiguous bases, and chimeric reads were removed, and paired-end reads were merged based on overlapping regions. Bacterial communities were analyzed by targeting the V3–V4 regions of the 16S rRNA gene, while fungal communities were analyzed by targeting the internal transcribed spacer (ITS) region. Sequencing libraries were prepared following the Illumina 16S and ITS Metagenomic Sequencing Library Preparation protocols [32]. For bacterial community analysis, PCR amplification of the 16S rRNA gene V3–V4 region was performed using the universal primer pair 16S V3-F (5-TCGTCGGCAGCGTCAGATGTGTATAAGAGACAGCCTACGGGNGGCWGCAG-3′) and 16S V4-R (5′-GTCTCGTGGGCTCGGAGATGTGTATAAGAGACAGGACTACHVGGGTATCTAATCC-3′), containing Illumina adapter overhang sequences. The first PCR consisted of an initial denaturation at 95 °C for 3 min, followed by 25 cycles of 95 °C for 30 s, 55 °C for 30 s, and 72 °C for 30 s, with a final extension at 72 °C for 5 min. For fungal community analysis, the ITS region was amplified using the ITS3 forward primer (5′-TCGTCGGCAGCGTCAGATGTGTATAAGAGACAGGCATCGATGAAGAACGCAGC-3′) and the ITS4 reverse primer (5′-GTCTCGTGGGCTCGGAGATGTGTATAAGAGACAGTCCTCCGCTTATTGATATGC-3′), following the same PCR conditions as described above. The first-round PCR products were purified using AMPure beads (Agencourt Bioscience, Beverly, MA, USA). Purified amplicons were subjected to index PCR using Nextera XT index primers (Illumina Inc., San Diego, CA, USA), with 10 cycles under the same PCR conditions. The indexed PCR products were purified again using AMPure beads. Library quality was assessed using a TapeStation D1000 ScreenTape system (Agilent Technologies, Waldbronn, Germany), and library quantification for sequencing was performed by quantitative PCR using KAPA Library Quantification Kits for Illumina Sequencing Platforms [33]. Paired-end sequencing (2 × 300 bp) was performed on the Miseq™ platform (Illumina, San Diego, CA, USA). Sequence data were processed using a standardized bioinformatics pipeline provided by the sequencing service provider (Macrogen, Sejong, Republic of Korea). Raw reads were preprocessed using Cutadapt (v3.2) to remove adapter and primer sequences [34]. Quality-filtered reads were denoised, merged, and chimera-checked using the DADA2 pipeline (v1.18.0) to generate amplicon sequence variants (ASVs) [35]. Taxonomic assignment was conducted using a Naïve Bayesian classifier against reference 16S and ITS databases, and normalization for microbial community comparison was performed by subsampling to the lowest sequencing depth using QIIME (v1.9). Multiple sequence alignment of ASVs was conducted using MAFFT (v7.475), and phylogenetic trees were constructed using FastTreeMP (v2.1.10) [36,37]. Alpha-diversity was evaluated using the Shannon diversity index based on amplicon sequence variants. The adequacy of sequencing depth for alpha-diversity estimation was assessed using rarefaction analysis.

### 2.4. pH Measurement During Kimchi Fermentation

The pH of kimchi samples was measured to evaluate acidification patterns during fermentation and their association with changes in microbial community structure. Kimchi was selected for pH analysis because lactic acid bacteria-driven fermentation plays a central role in kimchi production, and rapid pH reduction is a key determinant of microbial succession and fermentation dynamics. pH measurements were conducted for kimchi products, excluding aged kimchi, specifically cabbage kimchi and radish kimchi, and were performed separately for traditionally and commercially produced samples. pH was measured at the time of sampling (week 0) and subsequently at weekly intervals for a total fermentation period of four weeks (weeks 1–4). Prior to measurement, kimchi samples were thoroughly homogenized to ensure uniformity. The pH of the homogenized samples was measured using a calibrated pH meter (Orion Star A211; Thermo Fisher Scientific, Waltham, MA, USA). All pH measurements were performed in triplicate, and the mean values were calculated.

### 2.5. Statistical Analysis

Statistical analysis was performed only for pH measurements obtained during kimchi fermentation. Differences in pH values between traditionally and commercially produced kimchi were analyzed at each fermentation time using Student’s *t*-test with SAS software (version 9.4; SAS Institute, Cary, NC, USA). Statistical significance was defined at *p* < 0.05. Microbial community data, including Shannon diversity indices, were analyzed descriptively. Because amplicon sequencing was conducted once per pooled sample, statistical comparisons of microbial diversity indices between groups were not performed.

## 3. Results and Discussion

### 3.1. Microbial Succession During Fermentation of Cabbage and Radish Kimchi Prepared by Traditional and Commercial Methods

#### 3.1.1. Cabbage Kimchi

**Figure 1:**
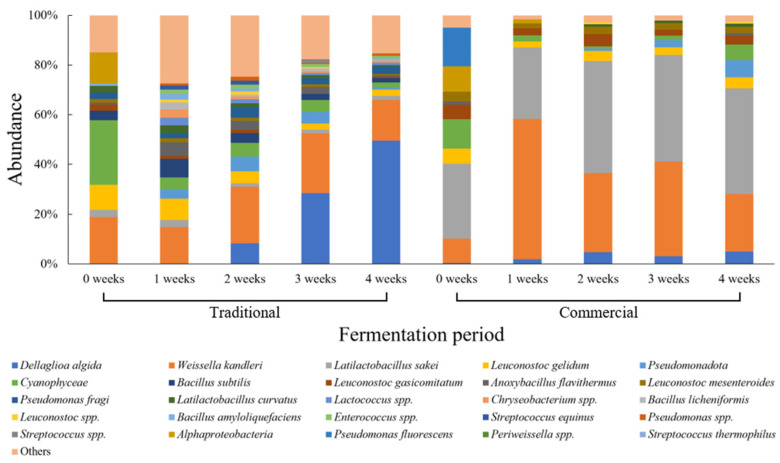
Relative abundance of microbial communities in traditional and commercial cabbage kimchi during fermentation.

Figure 1 shows the changes in microbial community composition of traditionally and commercially produced cabbage kimchi during fermentation. In traditionally produced kimchi, the microbial community was highly diverse at the early stage of fermentation. *Weissella kandleri* (18.9%) and *Leuconostoc gelidum* (10.1%) were the dominant taxa at week 0, while other lactic acid bacteria were detected at relatively low abundances. The microbial community remained diverse until week 2, with *W. kandleri* and *L. gelidum* persisting as the predominant taxa. After week 3 of fermentation, a clear shift in community structure was observed, with *Dellaglioa algida* (28.4%) emerging as the dominant taxon. By week 4, *D. algida* (49.5%) accounted for nearly half of the microbial community, while *W. kandleri* (16.5%) remained the second most abundant taxon. All other taxa were maintained at low relative abundances throughout the later fermentation period. In contrast, the initial microbial community of commercially produced cabbage kimchi differed markedly from that of traditionally produced kimchi. At week 0, *Latilactobacillus sakei* (30.3%) was the dominant taxon, whereas *W. kandleri* and *L. gelidum* were present at lower relative abundances. At week 1, *W. kandleri* (56.5%) rapidly increased and became the predominant taxon, resulting in a simplified community structure dominated by two taxa. This simplified microbial community, dominated by *L. sakei* and *W. kandleri*, persisted through week 4 of fermentation. Unlike traditionally produced kimchi, commercially produced kimchi exhibited a relatively stable and less diverse microbial community structure throughout fermentation.

The microbial succession patterns appeared to differ between traditionally and commercially produced cabbage kimchi. These observations suggest that product category, as defined by additive status and market distribution, may be associated with differences in fermentation ecosystems. However, in addition to product categorization, factors such as variability in raw material composition, salting practices, and storage temperature may also contribute to differences in microbial succession patterns observed during kimchi fermentation [38,39,40]. The early dominance of *W. kandleri* and *L. gelidum* in traditionally produced cabbage kimchi reflects the strong influence of indigenous microorganisms originating from raw ingredients, as previously reported [41]. As fermentation progresses, progressive acidification imposes strong selective pressure on the microbial community [38]. Under these conditions, acid-tolerant taxa are favored, whereas acid-sensitive microorganisms are gradually excluded. This selective process contributes to a gradual reduction in microbial diversity during the later stages of fermentation. In commercially produced cabbage kimchi, the relatively simplified community structure observed during fermentation may reflect a combination of controlled processing conditions, formulation characteristics, and potential additive effects rather than inoculation status alone. Although starter culture information was obtained from manufacturers and product labels, starter use was not consistently associated with product category. Therefore, the dominance of *L. sakei* in some products should not be interpreted solely as evidence of starter-driven fermentation. *L. sakei* is well adapted to low-temperature and acidic environments and contributes to flavor development through protein and amino acid degradation [42,43,44]. In addition, the presence of declared preservatives or acid regulators in certain retail products may influence early microbial selection by modifying initial pH conditions or exerting selective pressure on susceptible taxa [45]. Weak-acid preservatives are known to inhibit sensitive microorganisms under acidic conditions, potentially favoring more tolerant lactic acid bacteria and contributing to community simplification during storage and distribution [46]. Thus, the simplified community structure observed in commercially produced cabbage kimchi may be attributed to the strong acid tolerance of *L. sakei* and its rapid lactic acid production under these selective conditions.

#### 3.1.2. Radish Kimchi

**Figure 2:**
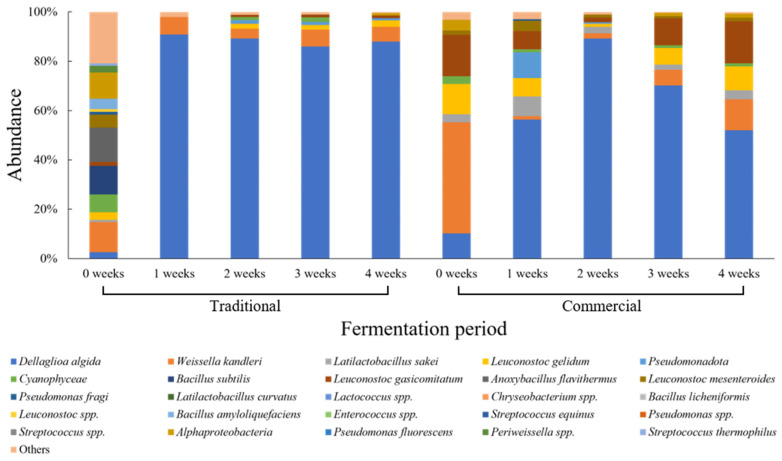
Relative abundance of microbial communities in traditional and commercial radish kimchi during fermentation.

Figure 2 shows the changes in microbial community composition of traditionally and commercially produced radish kimchi during fermentation. Traditionally produced radish kimchi exhibited high microbial diversity at week 0, with *W. kandleri* (12.2%) identified as the dominant taxon. After week 1, the microbial community composition shifted markedly, with *D. algida* (90.9%) rapidly increasing and becoming overwhelmingly dominant. This dominance was maintained through week 4 of fermentation, while the contributions of other lactic acid bacteria remained minimal. In contrast, the microbial community of commercially produced radish kimchi differed from that of traditionally produced radish kimchi. At the initial stage of fermentation, *W. kandleri* (45.0%) was the most abundant taxon, followed by *L. gelidum* (12.2%), *D. algida* (10.2%), and *L. sakei* (3.3%). At week 1, *D. algida* (56.3%) increased rapidly and became the predominant taxon. By week 2, *D. algida* effectively dominated the microbial community, whereas *W. kandleri* and *L. gelidum* were detected at low relative abundances. After week 3 of fermentation, the relative abundance of *D. algida* slightly decreased, while *W. kandleri* and *L. gelidum* partially recovered. By week 4, additional taxa, including *L. sakei*, increased in relative abundance. Overall, traditionally produced radish kimchi shifted from a diverse microbial community to dominance by a single taxon during fermentation, whereas commercially produced radish kimchi showed temporal changes in microbial community composition over time.

In radish kimchi fermentation, a pattern different from that observed in cabbage kimchi was identified. Traditionally produced radish kimchi rapidly transitioned from an initially diverse microbial community to dominance by a single taxon as fermentation progressed, primarily associated with the emergence of *D. algida*. This fermentation behavior may be attributed to differences in raw material characteristics between radish and cabbage [41]. Variations in water content, nutrient composition, and salting practices are known to influence fermentation dynamics and microbial community development, leading to substrate-specific fermentation trajectories [41,47,48]. In contrast, commercially produced radish kimchi exhibited lower microbial diversity at the initial stage but showed partial diversification during the later stages of fermentation. This U-shaped pattern may reflect a temporal restructuring of the microbial community during fermentation and storage. In addition to differences in processing conditions, formulation characteristics, including the possible presence of additives in certain commercial products, may contribute to early microbial selection [46], which could influence subsequent shifts in community composition over time.

### 3.2. Comparative Microbial Communities in Other Fermented Foods Prepared by Traditional and Commercial Methods

#### 3.2.1. Soy Sauce

**Figure 3:**
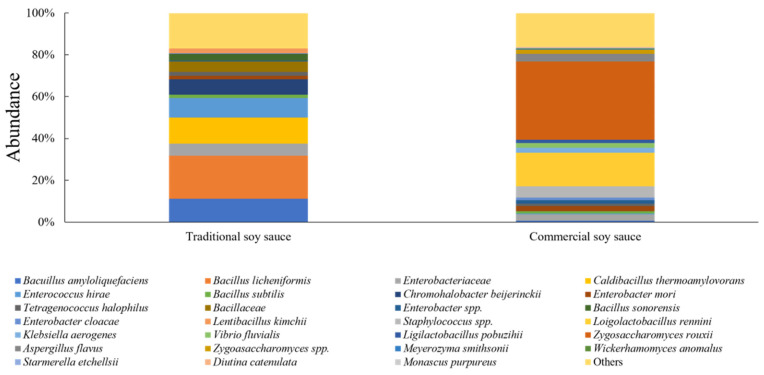
Relative abundance of microbial communities in traditionally and commercially produced soy sauce.

Figure 3 shows the microbial community composition of traditionally and commercially produced soy sauce. In soy sauce, traditionally produced soy sauce exhibited a more diverse microbial composition compared with commercially produced products. Traditionally produced soy sauce was dominated by *Bacillus* spp., with *Bacillus licheniformis* (20.5%) as the most abundant taxon, along with other halophilic bacteria, forming a complex microbial community structure. In contrast, commercially produced soy sauce exhibited a simplified microbial community dominated by yeasts and lactic acid bacteria. *Zygosaccharomyces rouxii* was the predominant taxon, followed by *Loigolactobacillus rennini*. Several additional bacterial and fungal taxa were detected at low relative abundances. Notably, *B. licheniformis* and *Caldibacillus thermoamylovorans*, which were abundant in traditionally produced soy sauce, were not detected in commercially produced soy sauce.

Traditionally produced soy sauce was characterized by the predominance of *Bacillus* spp., including *B. licheniformis* and *B. amyloliquefaciens*, which are known to produce proteases and α-amylase that hydrolyze soybean proteins and starches [49,50,51]. These enzyme activities supply amino acids and sugars during fermentation and are likely associated with the development of umami taste and roasted soybean-derived flavor attributes. In contrast, *Bacillus* spp. were not dominant in commercially produced soy sauce. Instead, the yeast *Z. rouxii* predominated, which is consistent with previous studies reporting its selective dominance under high-salinity fermentation conditions and its contribution to soy sauce flavor formation through the production of aromatic metabolites such as esters and aldehydes [52,53]. Traditionally produced soy sauce is derived from meju, where prolonged natural fermentation allows *Bacillus* spp. to establish and shape the fermentation ecosystem [54]. By contrast, commercially produced soy sauce is typically manufactured under high-salt conditions, which selectively favor salt-tolerant yeasts such as *Z. rouxii* [55,56]. As a result, traditionally produced soy sauce exhibited higher bacterial diversity dominated by *Bacillus* spp., whereas commercially produced soy sauce showed bacterial diversity associated with fungal dominance, despite retaining the capacity for flavor compound production.

#### 3.2.2. Soybean Paste and Pepper Paste

**Figure 4:**
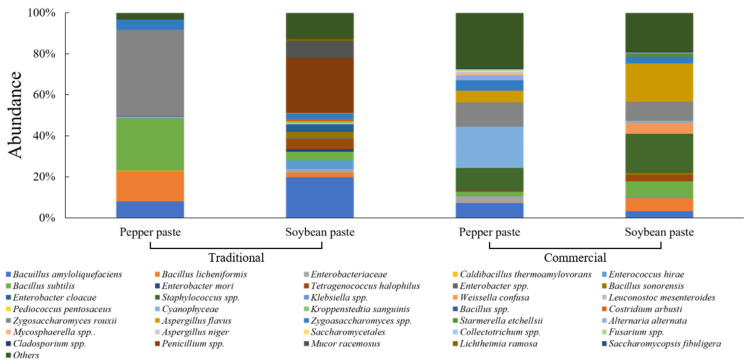
Relative abundance of microbial communities in traditionally and commercially produced soybean paste and pepper paste.

Figure 4 shows the microbial community composition of traditionally and commercially produced soybean paste and pepper paste. Traditionally produced soybean paste exhibited a mixed microbial community composed of *Bacillus* spp. and fungal taxa, with *Penicillium* spp. (26.8%) and *Bacillus* spp. (23.9%) as major contributors. In contrast, commercially produced soybean paste was dominated by different bacterial and fungal taxa, while *Bacillus* spp. were present at substantially lower relative abundances compared with traditionally produced soybean paste.

Traditionally produced soybean paste formed a complex microbial community in which *Bacillus* spp. coexisted with fungal and yeast taxa. This community structure reflects the characteristics of natural meju-based fermentation using cereals and soybeans [47]. Traditional meju fermentation has been reported to support the simultaneous proliferation of *Bacillus* spp. and fungi derived from raw materials and the surrounding environment, leading to the degradation of proteins and starches [57]. The coexistence of *Bacillus* spp. and fungal taxa is likely associated with the formation of low-molecular-weight compounds, including amino acids, thereby contributing to umami development [58]. In contrast, commercially produced soybean paste exhibited a microbial community dominated by a limited number of taxa. This pattern is consistent with the strong dependence of commercial processing on standardized starter cultures, high-salinity conditions, and relatively short fermentation periods, which collectively favor specific salt-tolerant taxa and reduce overall bacterial diversity [55,56,59].

Traditionally produced pepper paste exhibited a microbial community co-dominated by *Bacillus* spp. and yeasts, with *Z. rouxii* (42.1%) as the most abundant taxon. *Bacillus* spp. constituted a substantial proportion of the community, whereas minor taxa contributed at low relative abundances. In contrast, commercially produced pepper paste showed a more diverse microbial community with a reduced contribution of *Bacillus* spp. The community was characterized by a broader distribution of fungal and bacterial taxa and a markedly higher proportion of minor taxa. Overall, commercially produced pepper paste exhibited greater fungal diversity and a more heterogeneous microbial community structure than traditionally produced pepper paste.

A similar microbial community pattern was observed in pepper paste. Traditionally produced pepper paste was characterized by the co-dominance of *Bacillus* spp. and *Z. rouxii*, reflecting the complex fermentation matrix derived from soybean-based substrates [60]. In contrast, commercially produced pepper paste showed a reduced contribution of *Bacillus* spp. and a broader distribution of other bacterial and fungal taxa. This shift may be associated with differences in formulation and processing strategies applied in commercial products, which can influence microbial selection during fermentation and storage. Notably, *Staphylococcus* spp. and *Aspergillus flavus* were detected in commercially produced soybean paste and pepper paste. These microorganisms are known to include strains capable of producing enterotoxins and aflatoxins, respectively [61,62]. While toxin production was not evaluated in this study, the detection of these taxa highlights the importance of careful microbial monitoring during industrial fermentation processes, particularly when relying on limited starter systems.

In addition to differences in individual bacterial and fungal taxa, the coexistence of bacteria and fungi within soy-based fermented foods suggested potential interactions that may influence fermentation dynamics. Previous studies have reported that bacteria–fungi interactions can affect substrate utilization, metabolite production, and microbial succession in fermented food ecosystems [63]. In particular, the coexistence of *Bacillus* spp. and fungal taxa during traditional meju-based fermentation has been associated with protein and starch degradation, contributing to the formation of low-molecular-weight compounds [64]. Differences in food matrices and production strategies may therefore shape not only individual microbial populations but also the structure of coexisting bacterial and fungal communities.

#### 3.2.3. Other Fermented Foods

**Figure 5:**
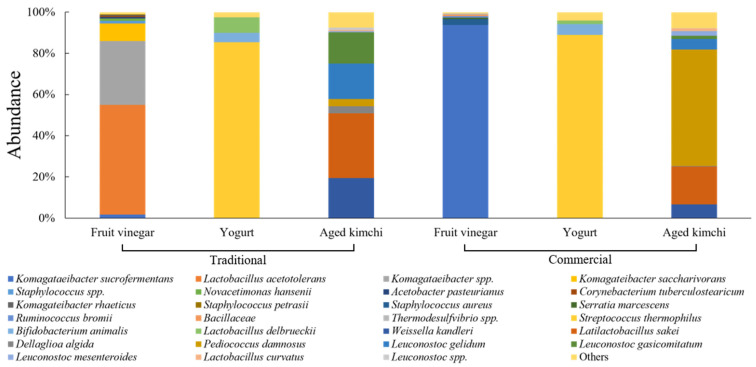
Relative abundance of microbial communities in traditionally and commercially produced fruit vinegar, yogurt, and aged kimchi.

Figure 5 shows the microbial community composition of fruit vinegar, yogurt, and aged kimchi produced using traditional and commercial methods. The microbial community distribution of fruit vinegar differed markedly according to the production method. Traditionally produced fruit vinegar exhibited a diverse assemblage of acetic acid bacteria, whereas commercially produced fruit vinegar was dominated by a single *Komagataeibacter* spp., including strong microbial simplification under industrial production.

In fruit vinegar, differences in microbial community diversity according to production method were more pronounced than those observed in other fermented foods. Traditionally produced fruit vinegar exhibited a complex microbial community composed of diverse acetic acid-producing bacteria, including *Komagataeibacter* spp. and *Lactobacillus acetotolerans*. In contrast, commercially produced fruit vinegar showed a simplified community structure dominated by *Komagataeibacter sucrofermentans*. These differences likely reflect contrasting fermentation strategies. Traditional fruit vinegar relies on spontaneous fermentation, which allows multiple microbial taxa to coexist and compete [65]. By contrast, commercial fruit vinegar production is generally conducted under tightly controlled acidification conditions aimed at rapid acidification and product consistency [66]. Because *Komagataeibacter* species exhibit high tolerance to elevated acetic acid concentrations, they are frequently associated with industrial acetification systems [67,68]. In addition, formulation and processing characteristics in commercially produced vinegar, including differences in ingredient composition and possible additive use, may further influence microbial selection during fermentation and storage, contributing to the simplified microbial profiles observed.

For yogurt, both traditionally and commercially produced products showed highly similar microbial communities dominated by *Streptococcus thermophilus*, with minor contributions from other lactic acid bacteria. This pattern indicates that yogurt microbiota were relatively stable regardless of production method, reflecting the standardized use of starter cultures.

No discernible differences in the microbial community structure of yogurt were observed between production methods. This uniformity reflects the inherent reliance of yogurt fermentation on standardized starter cultures [69]. *S. thermophilus* contributes to yogurt texture through milk protein coagulation [70]. The mutualistic exchange of nutrients and metabolites between *L. delbrueckii* represents a core mechanism underlying yogurt fermentation [71]. Consequently, yogurt shows minimal variation in microbial community structure according to production method.

Aged kimchi exhibited clear differences in microbial community composition between production methods. Traditionally produced aged kimchi was characterized by diverse lactic acid bacteria, whereas commercially produced aged kimchi showed pronounced dominance of *Pediococcus damnosus* and reduced lactic acid bacteria diversity. Overall, commercial production of aged kimchi was associated with a simplified microbial community structure dominated by specific taxa.

Aged kimchi is a long-term fermented product in which microbial communities generally become simplified during prolonged storage [72]. In the present study, traditionally produced aged kimchi maintained a complex community dominated by diverse lactic acid bacteria, including *L. sakei*, *L. gelidum*, and *W. kandleri*. In contrast, commercially produced aged kimchi was characterized by the pronounced dominance of *P. damnosus*. Previous studies have reported that lactic acid bacterial diversity in aged kimchi decreases over time, ultimately leading to *Pediococcus dominance* [72]. However, traditionally produced kimchi undergoes successive replacement among multiple lactic acid bacteria, which helps maintain microbial balance during long-term fermentation [73]. The dominance of *P. damnosus* in commercially produced aged kimchi may therefore reflect selective pressures associated with prolonged acidification and storage environments. Differences in processing control, ingredient composition, and possible additive use may further influence microbial succession patterns during aging, potentially contributing to the simplified community structure observed in certain commercial products.

### 3.3. Shannon Diversity Indices in Traditional and Commercial Fermented Foods

**Figure 6:**
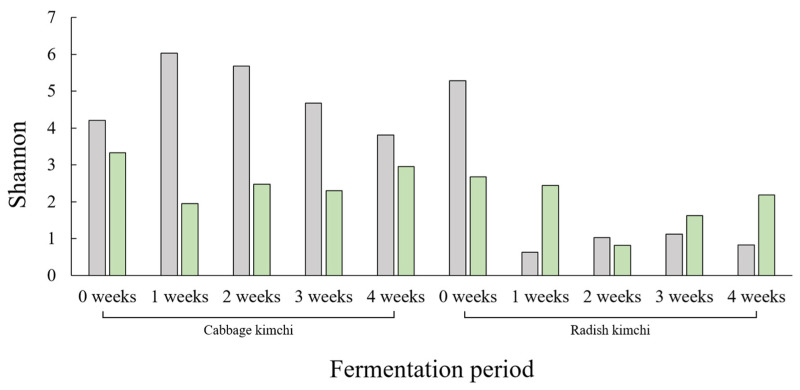
Shannon diversity of microbial communities in cabbage kimchi and radish kimchi during a four-week fermentation period. Gray and green bars represent traditionally and commercially produced products, respectively.

Figure 6 shows Shannon diversity indices of traditionally and commercially categorized kimchi during the four-week fermentation period. Because microbial community analysis was conducted on pooled samples, Shannon diversity values are presented descriptively and were not subjected to statistical comparison. In traditionally produced cabbage kimchi, Shannon diversity increased from 4.21 at 0 to 6.03 at week 1 and then decreased to 3.81 by week 4. In commercially produced cabbage kimchi, values decreased from 3.33 at week 0 to 1.95 at week 1, followed by values ranging from 2.30 to 2.95 through week 4. For radish kimchi, traditionally produced radish kimchi showed a value of 5.92 at week 0, decreasing to 0.62 at week 1 and remaining ≤1.12 thereafter. Commercially produced radish kimchi showed values of 2.68 at week 0, 0.82 at week 2, and 2.18 at week 4. These values illustrate distinct temporal patterns in Shannon diversity during fermentation.

**Figure 7:**
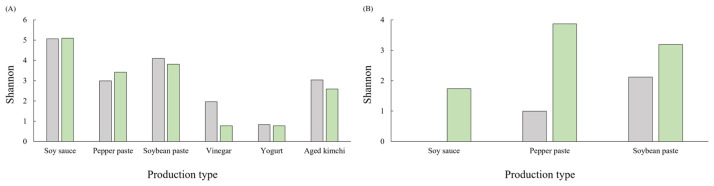
Shannon diversity of (**A**) bacterial and (**B**) fungal communities in fermented foods other than kimchi, according to production method. Gray and green bars represent traditionally and commercially produced products, respectively.

Figure 7 shows Shannon diversity indices of microbial communities in fermented foods other than kimchi. As with kimchi, microbial community analysis was performed on pooled samples; therefore, diversity values are presented descriptively to characterize relative patterns rather than to support inferential statistical comparisons. Diversity values varied according to food type. In soy sauce, Shannon diversity values were 5.07 and 5.10 for traditionally and commercially produced products, respectively. Soybean paste showed values of 4.10 and 3.82, while pepper paste showed values of 2.99 and 3.42 for traditionally and commercially produced products, respectively. Fruit vinegar and yogurt showed comparatively low values across both categories. Aged kimchi showed values of 3.03 and 2.59 for traditionally and commercially produced products. Fungal community Shannon diversity values also varied among food types. No fungal diversity was detected in traditionally produced soy sauce (0.00), whereas commercially categorized soy sauce showed a value of 1.74. In soybean paste and pepper paste, fungal diversity values were 2.12 and 1.00 for traditionally produced products and 3.19 and 3.87 for commercially produced products, respectively.

### 3.4. pH Changes During the Fermentation of Cabbage Kimchi and Radish Kimchi

**Figure 8:**
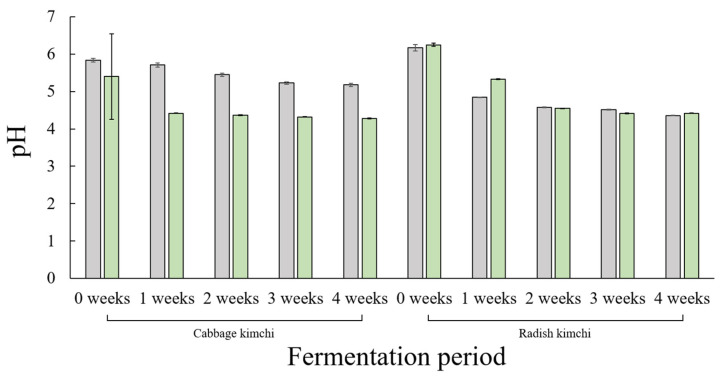
pH values of cabbage kimchi and radish kimchi during a four-week fermentation period. Gray and green bars represent traditionally and commercially produced products, respectively. Bars indicate standard deviations.

Figure 8 shows changes in pH values of cabbage kimchi and radish kimchi during the four-week fermentation period under traditional and commercial production. For traditionally produced cabbage kimchi, the initial pH at week 0 was 5.84 and gradually decreased to 5.71, 5.45, 5.23, and 5.18 at weeks 1, 2, 3, and 4, respectively. The decrease in pH was relatively gradual, and the pH remained above 5.0 even after four weeks of fermentation. In contrast, commercially produced cabbage kimchi exhibited a greater and more rapid decrease in pH. The initial pH was 5.40 at week 0 and declined to 4.42, 4.37, 4.32, and 4.28 at weeks 1, 2, 3, and 4, respectively. Accordingly, commercially produced cabbage kimchi acidified more rapidly and maintained consistently lower pH values throughout fermentation compared with traditionally produced cabbage kimchi.

For traditionally produced radish kimchi, the initial pH was relatively high (6.17) but decreased steadily during fermentation, reaching 4.36 by week 4. Commercially produced radish kimchi showed a similar overall decreasing trend, with pH declining from 6.25 at week 0 to 4.42 by the end of fermentation. Compared with traditionally produced radish kimchi, commercially produced radish kimchi exhibited more rapid acidification between weeks 0 and 2; however, both products reached similar pH levels by weeks 3 and 4.

Differences in microbial community structure during kimchi fermentation were closely associated with pH dynamics. Traditionally produced cabbage kimchi exhibited gradual acidification, whereas commercially produced cabbage kimchi showed a more rapid pH decline and consistently lower pH values. Rapid acidification in commercial fermentation can enhance microbial safety but may simultaneously reduce microbial diversity and flavor complexity [74,75,76,77]. In radish kimchi, both production methods reached similar final pH levels; however, early-stage acidification proceeded more rapidly in commercially produced products. This accelerated pH decrease is attributable to starter-dominated lactic acid bacteria and rapid organic acid accumulation, primarily lactic acid and acetic acid [78]. In commercially produced kimchi, the dominance of *L. sakei* favors homolactic fermentation, resulting in rapid acidification [73,79]. By contrast, traditionally produced kimchi is characterized by the coexistence of heterofermentative lactic acid bacteria, including *Leuconostoc* spp. and *Weissella* spp., which generate diverse metabolites and slow the rate of acidification [25,27]. As fermentation progresses, microbial succession toward acid-tolerant taxa leads to continued pH reduction. Overall, these results indicate that microbial succession governs acidification dynamics and that the acidification rate is a critical determinant of kimchi fermentation quality.

This study evaluated the effects of production methods on fermentation ecosystems by integrating species-level microbial community structure, Shannon α-diversity, and pH changes in traditionally and commercially produced Korean fermented foods. Fermented foods develop their characteristic sensory attributes and functional properties through microbial growth and metabolite production [1,80]. These properties can vary substantially depending on the production method [1,80,81]. In our study, traditionally categorized fermented foods generally exhibited more complex microbial community structures, whereas many commercially available products showed relatively simplified profiles. Rather than attributing these differences to a single production variable, the observed patterns likely reflect combined influences of fermentation environment, ingredient composition, additive usage, and formulation practices. Commercially produced fermented foods are often manufactured under more standardized conditions, including controlled salinity, fermentation duration, and the use of certain food additives such as preservatives or acidity regulators [82]. Such factors may impose selective pressures on microbial communities, potentially favoring adapted taxa and contributing to reduced overall diversity [83]. In contrast, products categorized as traditionally produced in this study were defined based on the absence of declared food additives and non-industrial production characteristics. Reduced preservative-based selection pressures and greater environmental variability may allow more heterogeneous microbial assemblages to persist [84]. Therefore, differences observed between production categories should be interpreted as multifactorial ecological outcomes rather than as the effect of a single mechanistic factor.

This study has methodological limitations. Microbial community analyses were performed on pooled samples, which limited the ability to conduct statistical comparisons of diversity indices between production methods. In addition, because traditionally produced samples were obtained from selected producers, the findings should be interpreted as reflecting general trends rather than fully representing all regional traditional fermented foods in Korea. Nevertheless, the integrated analysis of microbial community composition, α-diversity, and pH dynamics provides meaningful insights into how production methods shape fermentation ecosystems.

## 4. Conclusions

This study provides a comparative overview of microbial community patterns in traditionally and commercially produced Korean fermented foods across multiple food types, including kimchi, soybean-based products, vinegar, and yogurt. By examining community composition, α-diversity, and pH changes, the results highlight overall patterns of differences in microbial community structure associated with production methods across diverse fermentation matrices. Rather than focusing on a single food product, this multi-food approach offers broader insight into how variations in ingredient composition, processing control, and formulation characteristics, including potential additive use, are associated with differences in microbial community organization across diverse fermentation matrices. The characteristics of these patterns varied depending on food type, suggesting that microbial structure reflects the combined influence of substrate, fermentation environment, and product formulation. Overall, this study provides a descriptive framework for comparing fermentation ecosystems across different foods and production strategies, providing baseline information that may support future studies aimed at linking microbial community structure with functional and industrially relevant fermentation attributes.

## Data Availability

Upon request, summary data, including taxonomic profiles of microbial communities as well as information on traditional and commercial product manufacturers and brands analyzed in this study, may be made available for reasonable academic or research purposes, subject to institutional review and approval. Researchers interested in accessing summary data or further details may contact the corresponding author.

## Supplementary Materials

The following supporting information can be downloaded at: https://www.mdpi.com/article/10.3390/foods15061010/s1, Table S1: Detailed production procedures, manufacturer information, and starter culture use of kimchi samples included in this study; Table S2: Detailed production procedures, manufacturer information, and starter culture use of soy sauce samples included in this study; Table S3: Detailed production procedures, manufacturer information, and starter culture use of soybean paste samples included in this study; Table S4: Detailed production procedures, manufacturer information, and starter culture use of pepper paste samples included in this study; Table S5: Detailed production procedures, manufacturer information, and starter culture use of yogurt samples included in this study; Table S6: Detailed production procedures, manufacturer information, and starter culture use of fruit vinegar samples included in this study.
